# Application of EEG in migraine

**DOI:** 10.3389/fnhum.2023.1082317

**Published:** 2023-02-17

**Authors:** Ning Zhang, Yonghui Pan, Qihui Chen, Qingling Zhai, Ni Liu, Yanan Huang, Tingting Sun, Yake Lin, Linyuan He, Yue Hou, Qijun Yu, Hongyan Li, Shijiao Chen

**Affiliations:** ^1^Department of Neurology, The First Affiliated Hospital of Harbin Medical University, Harbin, Heilongjiang, China; ^2^Shanxi Bethune Hospital, Shanxi Academy of Medical Sciences, Tongji Shanxi Hospital, Third Hospital of Shanxi Medical University, Taiyuan, China; ^3^Tongji Medical College, Tongji Hospital, Huazhong University of Science and Technology, Wuhan, China

**Keywords:** migraine, EEG, spectrum power, microstates, functional connectivity, brain network, machine learning

## Abstract

Migraine is a common disease of the nervous system that seriously affects the quality of life of patients and constitutes a growing global health crisis. However, many limitations and challenges exist in migraine research, including the unclear etiology and the lack of specific biomarkers for diagnosis and treatment. Electroencephalography (EEG) is a neurophysiological technique for measuring brain activity. With the updating of data processing and analysis methods in recent years, EEG offers the possibility to explore altered brain functional patterns and brain network characteristics of migraines in depth. In this paper, we provide an overview of the methodology that can be applied to EEG data processing and analysis and a narrative review of EEG-based migraine-related research. To better understand the neural changes of migraine or to provide a new idea for the clinical diagnosis and treatment of migraine in the future, we discussed the study of EEG and evoked potential in migraine, compared the relevant research methods, and put forwards suggestions for future migraine EEG studies.

## 1. Introduction

Migraine is a complex brain disease affecting more than 1 billion people worldwide and is the main cause of disability in the world population. In the past three decades, the global incidence rate of migraine has increased significantly, resulting in serious disease and economic burdens (Amiri et al., 2021; Ashina et al., 2021; Safiri et al., 2022). The International Headache Association defines migraine as a recurrent primary headache disorder that lasts for 4–72 h. Generally, headache is single, pulsatile, moderate or severe, aggravated by routine physical activity, and accompanied by nausea, photophobia, and hydrophobia (Headache Classification Committee of the International Headache Society [IHS], 2013). Approximately one-third of migraine attacks are preceded by a precursor (Noseda and Burstein, 2013). The most common aura symptom is visual impairment, and other common symptoms include sensory, language and motor disorders, as well as high-level cortical dysfunction (Eriksen et al., 2004).

The pathophysiology of migraine is complex (Dodick, 2018), and still not completely understood. There is a lack of specific biomarkers, and the diagnosis mainly depends on clinical manifestations. In the past 10 years, an increasing number of studies have begun to explore the specific biomarkers of migraine and its pathogenesis. A number of studies support that the cerebral cortex is the key layer of migraine (Barbanti et al., 2020) and that the biological matrix of migraine aura is a cortical electrical activity event called cortical spreading depression (CSD), which is related to a large number of transmembrane movements of multiple ions (Lai and Dilli, 2020). It follows that migraine involves the most high-level human motor–brain activity.

Electroencephalography (EEG) records the spontaneous and rhythmic electrical activity of the brain cell population and has become a powerful tool to explain the state of brain activity before the era of neuroimaging. Previously reported findings in migraine include slow activity, spike wave activity, reduced amplitude of background activity (Sand, 2003), hyperventilation response (Tan et al., 2007), and photic driving response (Bjork et al., 2011a). However, these clinical electrical studies mainly rely on traditional visual EEG analysis, and its application in the diagnosis of migraine has been controversial (Gronseth and Greenberg, 1995).

## 2. EEG signal processing and analysis methods

### 2.1. EEG signal preprocessing and feature extraction

Electroencephalography is a low-cost, non-invasive, and high-temporal resolution neuroelectrophysiological technology that has been widely used in medical fields (Briels et al., 2020; Furbass et al., 2021) and non-medical fields (Trejo et al., 2006; Lim et al., 2022). However, EEG signals are complex, high-dimensional (Hasenstab et al., 2017) and non-stationary and have the characteristics of a low signal-to-noise ratio in the time domain. Therefore, the application of EEG based on various methodologies requires the preprocessing of EEG signals. At present, the commonly used preprocessing methods include regression methods, blind source separation methods (BSS), wavelet transform (WT), filtering methods, etc. The three typical methods using the BSS algorithm are principal component analysis (PCA), independent component analysis (ICA), and canonical correlation analysis (CCA). The filtering methods include frequency filtering, adaptive filtering, and Wiener filtering.

**FIGURE 1:**
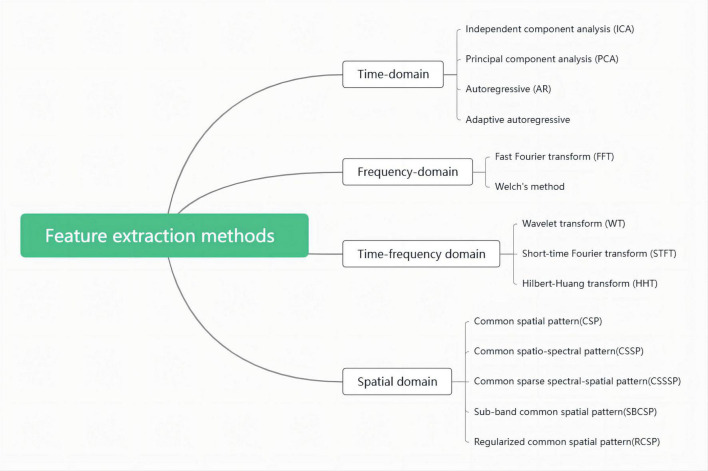
Feature extraction methods.

After preprocessing, the original EEG signal has removed all kinds of artifacts and noises and becomes a relatively pure EEG signal. However, due to the large amount of EEG data and the complexity of direct processing, feature extraction is also required to reduce the data dimension. These extraction methods include the time domain, frequency domain, time-frequency domain, and spatial information in the signal. These methods decompose the original EEG signals recorded from the scalp into activities at different frequencies compressed in the signal spectrum and then conduct quantitative analysis of activities in each frequency band, either at rest or under stimulation. Commonly used feature extraction methods are showed in Figure 1.

Each of the above methods has its advantages and disadvantages, which can be found in the following extensive review (Saeidi et al., 2021). Some methods run through the two stages of EEG signal preprocessing and feature extraction. In research, appropriate methods should be selected according to the specific nature of the research task.

### 2.2. Functional connectivity

**FIGURE 2:**
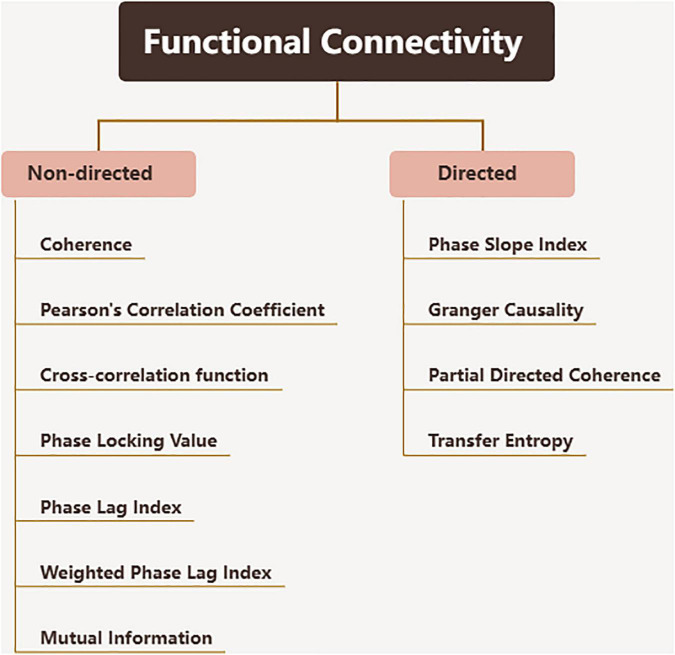
Functional connectivity metrics.

The complexity of brain function is based on the dynamic relationship between the cortex and subcortical regions, which enables the brain to adapt to different physiological and pathological conditions. The functional connectivity realizes the exploration of this dynamic relationship. Functional connectivity is defined as the temporal correlation between spatially distant neurophysiological events, expressed as the statistical independence deviation of these events between distributed neuron groups and regions (Lee et al., 2003; Fingelkurts et al., 2005). Effective connectivity is a relatively updated concept, defined as the direct or indirect influence exerted by one nervous system on another (Horwitz, 2003; Lee et al., 2003), which describes the dynamic directional interaction between brain regions. Since functional and effective connectivity technology largely depends on calculating the correspondence of neural signals over time, EEG with high temporal resolution is the best way to calculate such connectivity. The methods used to evaluate connectivity are very different (Sakkalis, 2011), and the calculation algorithms and concepts used to define brain connectivity values also vary widely between studies. More examples of problems can be found in the following review (Babiloni et al., 2020). Some conventional functional connectivity metrics are showed in Figure 2.

### 2.3. Brain network

Electroencephalography data can be used to construct a connection matrix and functional network through network analysis. Various complex methods are used in brain network research, such as EEG traceability analysis and graph theory analysis (Miraglia et al., 2017). Unlike connectivity research, which only provides information about how different brain regions (functions) connect, brain network research analyses the characteristics of brain networks. Complex network analysis is a branch of graph theory that simplifies the brain into a collection of “nodes” and “edges” and allows quantitative characterization of these networks (Rubinov and Sporns, 2010). Various global and local network metrics can be inferred from the networks. The functional network is based on the strength or consistency of functional interaction between network nodes. In a weighted network, the strength of such interaction is considered, while in an unweighted network, only the presence or absence of interaction is considered.

### 2.4. Source localization

**FIGURE 3:**
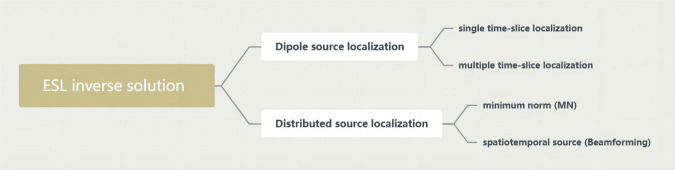
Inverse solution methods.

Electroencephalography source localization (ESL) demonstrates the synchronously activated neuronal populations underlying EEG activity by computing their cortical localization from the scalp distribution of the electric field. This is called solving the inverse problem of the EEG (Clemens et al., 2008). The analysis of neurophysiological signals in source space cannot completely overcome the problems of field diffusion and volume conduction. Therefore, it is suggested that source space analysis be combined with robust connectivity measurements to provide a variety of source space analysis methods, including low-resolution electromagnetic tomography (LORETA). Some of today’s most widely used inverse solution methods are in Figure 3.

### 2.5. Microstate analysis

Electroencephalography microstate analysis is a method to identify quasistable functional brain states, which reflects the transient and stable brain topology at the millisecond level. The transition between microstates can be interpreted as representing the sequential activation of different neural networks (Khanna et al., 2015), and EEG microstate analysis is designed to characterize these models using data compression or clustering techniques. Because this technique simultaneously considers signals recorded from all areas of the cortex, it is capable of assessing the function of large-scale brain networks.

### 2.6. Machine learning and deep learning

**FIGURE 4:**
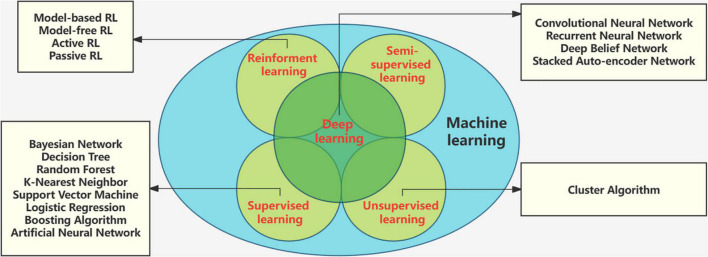
Methods and technologies of machine learning.

Machine learning (ML) is a rising research hotspot in the field of artificial intelligence. It abstracts human brain neural networks from the perspective of information processing, establishes corresponding models, and forms different networks according to different connection methods. It has been widely used in medical diagnosis, especially in the detection and analysis of biomedical signals. Electroencephalography based machine learning studies for diagnostic classification and tracking of therapeutic effects. Deep learning (DL) is a new branch of ML that has received widespread attention in EEG classification tasks (Saeidi et al., 2021). The root of DL techniques lies in the Artificial Neural Network (ANN). Unlike machine learning techniques, there is no need to extract features separately in deep learning, and the architectures support automatic feature extraction (Gautam and Sharma, 2020). Machine learning tools have developed rapidly. The methods and technologies of machine learning are in Figure 4.

**FIGURE 5:**
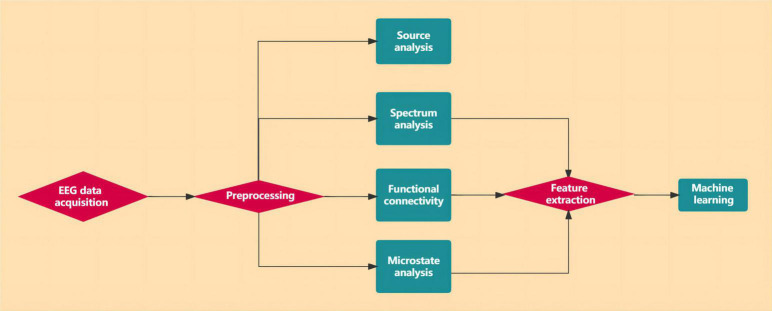
The flow chart of the EEG data analysis.

Figure 5 illustrates the flow chart of the EEG data analysis.

## 3. EEG study of migraine

### 3.1. Analysis of EEG spectrum power

**FIGURE 6:**
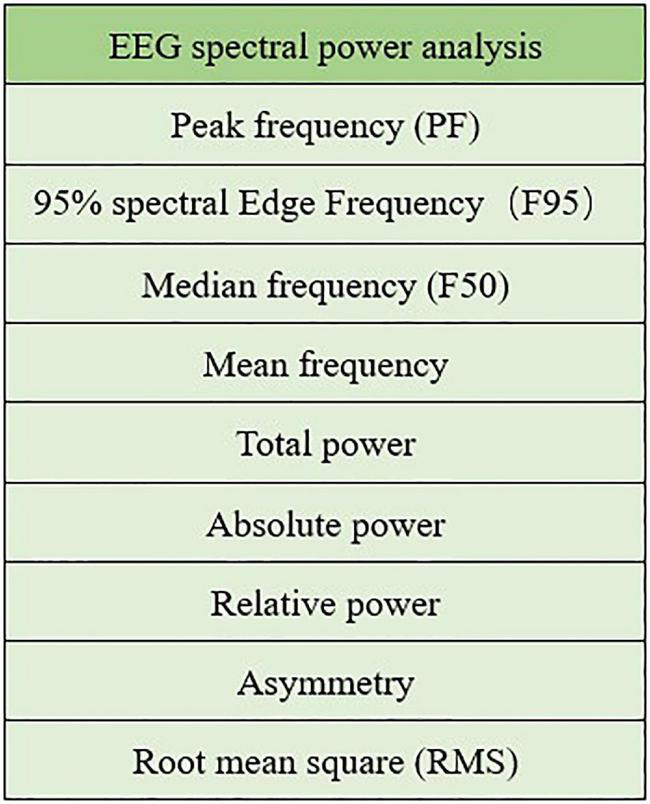
Spectral power analysis metrics.

A well-established and commonly used method to analyse EEG signals is spectral power analysis. The EEG spectrum mode is usually within the range of 0–30 Hz, and there are five internationally recognized frequency bands: delta (0–4 Hz), theta (5–7 Hz), alpha (8–13 Hz), beta (14–30 Hz), and gamma(>30 Hz). Some EEG spectral power analysis metrics are showed in Figure 6.

One study calculated the relative power of the four EEG frequency bands (delta, theta, alpha, and beta) to compare multiple groups of transient neurological deficit disorders, including migraine, and found a significant increase in the alpha relative power and a significant decrease in the beta relative power (Vellieux et al., 2021). Similarly, EEG spectral analysis was carried out in 20 untreated migraine without aura patients. It was found that except for F4 and C3, alpha power was higher than that of the control group; however, statistically significant spectral differences were found only in the right occipital region (Clemens et al., 2008). Other studies have yielded different results. The researchers analyzed the absolute power, relative power, and asymmetry of the delta, theta, and alpha frequency bands in parieto-occipital, temporal, and fronto-central areas and found globally increased relative theta activity in migraineurs (Bjork et al., 2009a).

Neurophysiological studies have shown fluctuating neurological dysfunction in migraine patients. The study analyzed alpha peak frequency, variability, peak power, and asymmetry in 41 migraine patients and 32 controls. The results showed that decreased peak frequency correlated with increased duration of disease and seizures. Frequency variability increases before onset, while peak power increases during episodes. Small changes in alpha rhythms were observed during the migraine cycle, indicating that the cumulative burden of migraine led to slight physiological changes in the optic cortex (Bjork et al., 2009b). Fluctuations in alpha oscillation (8–14 Hz) are considered to regulate visual perception. Spectral analysis of brain visual region alpha band oscillation (8–12 Hz) for observation in the migraine group and control group found that lower alpha band (8–10 Hz) power increased in migraine patients compared to controls (O’Hare et al., 2018).

In addition, the study divided migraine patients into episodic and chronic migraine subgroups and measured the resting-state EEG spectrum in different subgroups and healthy controls. Specific frequency bands were identified to distinguish the control group and the migraine group, as well as the migraine subgroup. The results showed that the frequency from 11.6 to 12.8 Hz was the overall feature of migraine, with significant differences between the central and left parietal lobe regions. The frequency band between 24.1 and 29.8 Hz was used to distinguish migraine subgroups and was found to have a positive correlation between the power of this band and time of onset in episodic migraine patients but not in chronic migraine patients (Gomez-Pilar et al., 2020).

Using standard EEG spectral analysis and event-related potential (ERP) methods, Martins et al. (2020) grouped migraineurs by different phases of the attack. The results showed that 24 h before the onset of headache, the relative power changes were statistically significant in the delta (decrease) and beta (increase) frequency bands, and this study confirms that EEG can detect neurophysiological changes before a migraine attack, making it possible for migraineurs to promptly treat an upcoming attack. In a retrospective analysis of EEG records of 40 patients with migraine at different periods, the power and power asymmetry at 36 and 72 h before and after the attack were calculated and compared with the interval value. The results suggest that migraineurs are most susceptible to attack when anterior quantitative electroencephalography (QEEG) delta power and posterior alpha and theta asymmetry values are high (Bjork and Sand, 2008). In another study, the author found slow and asymmetric EEG activity in migraineurs within 36 h before onset and found that higher theta power and photoresponse inhibition were associated with higher trigger sensitivity, the phenomenon of photophobia, and the severity of symptoms (Bjork et al., 2011b).

Hypersensitivity to sensory inputs is one of the core symptoms of migraine attacks, and migraineurs are hypersensitive to most sensory areas, such as visual, auditory, or somatosensory processing, even during interictal periods. The migraine brain processes sensory inputs such as auditory, olfactory, somatosensory, visual, and nociceptive stimuli differently than non-migraine populations (Moulton et al., 2011), which has been discussed to be due to hyperresponsiveness of primary sensory areas (Boulloche et al., 2010) or lack of habitual responses to repetitive stimuli (Magis et al., 2013).

Visual stimulation has proven to be a simple and reliable technique with the potential to detect cortical reactivity changes associated with migraine attacks. Quantitative EEG and steady-state visual evoked potentials (SSVEPs) at 27 Hz stimulation during the critical phase of migraine and in attack-free periods were examined in 16 patients with migraine without aura, and the main EEG abnormalities were slowing and asymmetry of the alpha frequency. It has been confirmed that fluctuating changes in alpha activity in migraine attacks may be associated with dysfunction (de Tommaso et al., 1998). Takashima et al. (2015) analyzed the EEG of migraine patients by rapid Fourier transform (FFT), observed the response of spectral peaks and topologies to light stimulation, and found that patients with longer disease duration were more likely to have higher global field power (GFP) values in driving responses. Another study included 28 migraine patients (17 with aura/11 without) and 29 non-migraine patients and compared the power of alpha during and before and after the visual stimulus and found that in migraineurs before stimulus onset, alpha power was significantly reduced relative to controls, indicating that migraine patients have alpha defects in the prophase of visual stimulation. Given that alpha activity is related to functional inhibition of the sensory cortex, the present study is consistent with the notion that migraine patients have a hyperresponsive visual cortex (Fong et al., 2022).

Migraine is associated with alterations in sensory processing and cortical reactivity, which may contribute to susceptibility to seizures by altering brain network excitability dynamics. Time frequency analysis and cross-correlation analysis by means of complex Morlet wavelets were performed to evaluate laser-induced changes in EEG and the most active cortical areas, and the results showed that the predictability of the sequence induced by laser stimulation was very different between controls and migraine patients, indicating that the cortex of migraine patients had an insufficient response to pain (de Tommaso et al., 2005). Studies of the physiological response to stress in migraine patients have shown that non-nociceptive mild stress does not produce any QEEG changes, but nociceptive stress leads to a significant decrease in alpha band power in all brain regions. Migraine patients exhibit a lower physiological stress threshold, confirming previous studies demonstrating that migraine is a disorder characterized by altered neural excitability (Rainero et al., 2001).

### 3.2. EEG-based studies of migraine-related functional connectivity and brain networks

The application of a functional connectivity algorithm provides support for the neural theory of migraine and opens up a new perspective for the functional connectivity disorder of the brain with normal structure. In recent years, various studies have compared the functional connectivity between different migraine groups.

One study used a customized ultrahigh-density EEG system to obtain the spatial coherence of each EEG band signal in migraine patients and comprehensively evaluated the changes in cortical coherence patterns during rest and sensory (visual and auditory) stimulation. The analysis showed that migraine patients showed low spatial coherence of alpha activity. In both sensory stimulation conditions as well as in the resting state, the decrease in consistency occurred predominantly in the frontal lobes, regardless of stimulation frequency. The abnormal pattern considering their EEG coherence may be associated with the cortical hyperreactivity of abnormal sensory processing in migraine patients (Chamanzar et al., 2021).

De Tommaso et al. investigated EEG signals by Morlet wavelets (MWT), synchrony entropy (SE), and Granger causality (GC) and mapped statistically validated results to corresponding scalp locations. To design a novel analysis of the effects of EEG synchronization and directional kinetics in patients with migraine versus healthy volunteers without migraine under pain stimulation. It was found that the vertex complexes of the averaged laser-evoked response (LEP) showed reduced habituation compared to the control group. Brain network analysis may help to understand the subtle changes in pain processes in migraine patients under laser stimulation (de Tommaso et al., 2015).

Using multichannel EEG recordings, transfer entropy analysis was conducted on the brain regions of migraine patients with aura and without aura and the control group under mode reversal visual stimulation to evaluate the subtle differences in brain networks between the two types of migraine. Granger causality results confirmed that although patients with aura have a special connection mode of the parieto-occipital cortex, the brains of the two subtypes respond to visual stimuli in the same way. Brain networking analysis showed that migraine without aura (MO) patients had increased intrahemispheric global efficiency with respect to the migraine with aura (MA), especially in frontal-central areas. At the same time, the MA showed a larger efficiency in sorting information from the left to the right parietal-occipital areas (higher interhemispherical efficiency) (de Tommaso et al., 2017). Under the same grouping mode, researchers chose a repetitive light stimulation paradigm to analyse functional and effective connectivity patterns. The results showed that the phase synchronization of the alpha band in the MO increased and the phase synchronization of the beta band in the MA decreased compared with the control group. Granger causality showed that compared to controls, the intensity of directional interactions in the beta band in MA patients increased compared to the control group. The apparent difference under visual stimulation between the two forms of migraine may be due to increased cortical activation in migraine with aura, as well as compensatory phenomena of reduced connectivity and functional network separation seen in patients without aura symptoms (de Tommaso et al., 2013). Similar to the above study groupings, subjects were found to exhibit lower interhemispheric coherence values (delta band) at C3–C4 when compared to controls by coherence analysis and exhibited lower coherence (beta band) at F3–F4 and C3–C4. Compared with subjects without visual aura, subjects with visual aura showed lower interhemispheric coherence (alpha band) at O1–O2 and T5–T6 (Koeda et al., 1999).

Studies have compared resting state EEG energy intensity and effective connectivity during different migraine phases between MO patients and healthy controls (HCs) using EEG power and coherence analysis. EEG effective connectivity in pre-ictal patients showed enhanced coupling of frontal-central and central-posterior networks and decreased coupling of frontal-occipital networks. Such brain network dynamics may contribute to the understanding of the complex neurophysiology before migraine attacks (Cao et al., 2016).

The variety of symptoms and neurological disorders observed during the various phases of migraine are complex and widespread, and the impairments in sensory, emotional, cognitive, and autonomic functions that may be experienced suggest that multiple neural networks are involved (Goadsby et al., 2017). EEG-based brain network studies have revealed differences in network properties between migraine patients and normal brains, explaining the fluctuation of brain states in different phases of migraine, the different phenotypes of aura perception, and the nature of pain processing.

### 3.3. Migraine-related EEG source localization analysis

Intracranial EEG source localization is commonly determined from low-resolution brain electromagnetic tomography (LORETA), and LORETA reveals the anatomical distribution of the cortical sources of EEG abnormalities in migraine.

Three studies used EEG to explore the dysfunctional regions of migraine by LORETA. The author of one study suggested that functional disturbance of the prefrontal cortex may play a potential role in pediatric migraine (Ouyang et al., 2020). Two other studies separately explored the light-driven response of EEG in migraine patients (Takashima et al., 2015) and its response to thermal stimuli (Lev et al., 2013), with the former finding that the limbic system may be involved in the central sensitization of the visual system.

### 3.4. Migraine-related EEG microstate analysis

Electroencephalography microstates are often referred to as global patterns of spatial configurations of electric potentials. The quasistable period of a single configuration is a kind of “microstate.” Although there are a large number of possible spatial configurations, they can generally be divided into four typical categories, labeled A–D. Microstates A–D corresponded to resting-state networks (RSNs) previously identified as associated with phonological processing, the visual network, the saliency network, and attention, respectively. The key characteristics of EEG microstates make it helpful to detect the dynamic activities in the resting-state brain network of nervous system diseases.

One study selected 61 MO patients (50 female) and 66 HC patients (50 female) for resting EEG to compare microstate parameters between the two groups. The results showed that compared with the control group, microstates B and D in the MO group showed higher time coverage and incidence, while microstate C showed lower time coverage and incidence, and the average duration was significantly shortened. Furthermore, in MO patients, the duration of microstate C was negatively correlated with clinical measures of headache-related disability as assessed by the six-item Headache Impact Test (HIT-6). This study has deepened the understanding of migraine pathophysiology by exploring the characteristics of EEG microstates at baseline, exploring the neurobiological mechanisms behind cortical excitatory changes and abnormal sensory, emotional and cognitive processing (Li et al., 2022).

### 3.5. EEG-based study of migraine-related machine learning

Different AI approaches including machine learning and deep learning are all based on the concept of developing prediction algorithms from large amounts of data, or big data. In this context, there are studies in the relevant literature that diagnose migraine using EEG signals and machine learning or other algorithms. Aslan et al. used a well-known integrated learning technique to classify the characteristic values obtained in each subband of the EEG signal and tested its classification performance for diagnosing migraine. The highest classification performance has been achieved with the Rotation Forest classifier for all evaluation metrics (Aslan, 2021). One study analyzed functional connectivity extracted from EEG signals acquired during interictal periods in 52 participants and tested using classifiers and found that the EEG functional connectivity obtained at rest could be used as an objective biomarker to distinguish migraine subgroups with good specificity and sensitivity (Frid et al., 2020). Classification models based on EEG have also been used to identify differences between the different phases of migraine. The complexity of the frontal EEG of migraine was identified using entropy-based analytical methods, and this reproducible complexity feature was applied for classification model testing and found that the Support vector machine radial basis function (SVM-RBF) classifier significantly outperformed other classifiers in classification accuracy, with the potential to provide pre-ictal alarms to patients without migraine aura (Cao et al., 2018).

Different DL techniques have been used to diagnose different neuropsychiatric and neurological disorders, but fewer articles have been published on migraine (Yang et al., 2018).

The summary of Resting-state EEG studies in migraine is shown in Table 1.

**TABLE 1:** Summary of Resting-state EEG studies in migraine.

References	Subjects	Migraine phase	Main methods	Main result
Clemens et al. (2008)	MO: 20 HC: 17	Inter-ictal	Spectral analysis LORETA	α power (↑)in all but two derivations (F4 and C3)
Bjork et al. (2009a)	MP: 33 HC: 31	Inter-ictal	Spectral analysis	relative θ activity (↑)
Bjork and Sand (2008)	MA: 8 MO: 33	Inter-ictal Pre-ictal Ictal Post-ictal	Spectral analysis	Migraineurs are most susceptible to attack when anterior δ power and posterior α and θ asymmetry values are high
Bjork et al. (2009b)	MA: 5 MO: 20 HC: 18	Inter-ictal Pre-ictal Ictal Post-ictal	Spectral analysis	Pre-ictal: α frequency variability (↑) Ictal: α peak power (↑)
Gomez-Pilar et al. (2020)	EM: 45 CM: 42 HC: 39	Inter-ictal	Spectral analysis	Distinguish MP and HC:11.6–12.8 Hz Distinguish EM and CM:24.1, 29.8 Hz
Koeda et al. (1999)	MA: 5 MO: 15 HC: 20	Inter-ictal	Coherence	ICoh (↓) MP vs. HC HCoh (↑) MP vs. HC ICoh (↓) MA vs. MO
Cao et al. (2016)	EM: 55 HC: 20	Inter-ictal Pre-ictal Ictal Post-ictal	Coherence	Inter-ictal, ictal: EEG power and coherence (↓) EM vs. HC; power density and EC differ between migraine phases
Ouyang et al. (2020)	MP: 40 HC: 40	Inter-ictal	LORETA	left prefrontal cortex FC(↑)MP vs. HC

δ, delta; θ, theta; α, alpha; β, beta; MP, migraine patients; MA, migraine with aura; MO, migraine without aura; CM, chronic migraine; EM, episodic migraine; HC, healthy controls; GFP, global field power. ICoh, interhemispheric coherence; HCoh, intrahemispheric coherence; FC, functional connectivity.

The summary of Stimulus-state EEG studies in migraine is shown in Table 2.

**TABLE 2:** Summary of Stimulus-state EEG studies in migraine.

References	Subjects	State	Migraine phase	Main methods	Main result
Bjork et al. (2011b)	MA: 8 MO: 33 HC: 32	Photic stimulation	Inter-ictal Pre-ictal ictal Post-ictal	Spectral analysis SSVEP	Inter-ictal: relative θ activity (↑) Pre-ictal: δ absolute power (↑) (frontocentral)
Martins et al. (2020)	MP: 24	Visual attention	Migraine cycle period	Spectral analysis ERP	Pre-ictal : relative power in the δ (↓) and β (↑)
de Tommaso et al. (1998)	MP: 16 HC: 20	Flash Stimulation (27 Hz)	Inter-ictal ictal	Spectral analysis SSVEP	Main spontaneous EEG abnormalities: the slowing and asymmetry of the dominant frequency in the α range.
Takashima et al. (2015)	MA: 11 MO: 17	Photic stimulation	Inter-ictal	GFP spectrum analysis	The GFP value had a positive correlation with the duration of illness.
de Tommaso et al. (2005)	MO: 10 HC: 7	Laser stimulation	Inter-ictal	Power spectral density LEP	The predictability of the series changed very differently in controls and patients.
Rainero et al. (2001)	MO: 19 HC1: 16 HC2: 14	Ischemic stress	Inter-ictal	Spectral analysis	The non-noxious stress:α power (↓)
O’Hare et al. (2018)	MP: 13 HC: 17	Visual task Noise task	Inter-ictal ictal	Spectral analysis	Lower alpha-band (8 to 10 Hz) power (↑)
Fong et al. (2022)	MA: 17 MO: 11 HC: 29	Visual attention	Inter-ictal	Spectral analysis	Pre-stimulus period:α Power (↓)
Bassez et al. (2020)	MO: 23 HC: 23	Laser stimulation	Inter-ictal	Spectral analysis DICS LEP	Increases in central GBOs were not significantly different MO vs. HC
Porcaro et al. (2017)	MO: 20 HC: 20	Electrical stimulation	Inter-ictal	FSS spectral analysis	BS and Th HFO activation Bilaterally (↓) MO vs. HC
Chamanzar et al. (2021)	MP: 14 HC: 18	Visual and auditory stimulation	Inter-ictal	Coherence spectral analysis	Spatial coherence of α-band activity (↓) in frontal clusters
de Tommaso et al. (2015)	MO: 31 HC: 19	Laser stimulation	Inter-ictal	MWT, SE, GC	Post-stimulus period: the same cortical areas were more connected MO vs. HC
de Tommaso et al. (2017)	MA: 19 MO: 19 HC: 11	Visual stimulation	Inter-ictal	GC, TE	Resting-state: information flow (↓) in MO vs. MA Inter-stimulus: a different information transfer (MO vs. MA, MP vs. HC)
de Tommaso et al. (2013)	MA: 19 MO: 19 HC: 11	Repetitive photic stimulation	Inter-ictal	Phase synchronization GC	Phase synchronization in α band (↑) MO vs. HC; in β band (↓) MA vs. HC; directed interactions in β band (↑)MA vs. MO, MA vs. HC
Bassez et al. (2020)	MO: 23 HC: 20	laser stimulation	Inter-ictal	LEPs DCM	After repetitive stimulations connection strengths: HC (↑) vs. MO

α, alpha; β, beta; MP, migraine patients; MA, migraine with aura; MO, migraine without aura; EM, episodic migraine; HC, healthy controls; MWT, Morlet wavelet; SE, synchronization entropy; GC, Granger causality; TE, transfer entropy; ICoh, interhemispheric coherence; HCoh, intrahemispheric coherence; EM, episodic migraine; EC, effective connectivity; DICS, Dynamic Imaging of Coherent Sources; GBOs, Gamma-band oscillations; FSS, functional source separation; BS, lower brainstem; Th, thalamic; HFO, high- frequency oscillatory; DCM,dynamic causal modelling.

## 4. Discussion

### 4.1. EEG features of migraine and the subtypes

The spectral characteristics and functional connectivity patterns of electrical brain activity in migraine are different. Changes in EEG frequency bands have been commonly observed. Compared with healthy controls, the main alpha band abnormalities found were the slowing and asymmetry of the dominant frequency (Nyrke et al., 1990; de Tommaso et al., 1998; O’Hare et al., 2018), different brain region alpha increases or decreases in activity (Clemens et al., 2008; Bjork et al., 2009a; Ojha and Panda, 2022), alpha oscillatory fluctuations, particularly on tasks relying on temporal integration (O’Hare et al., 2018), visual stimuli (Fong et al., 2022), and noxious/non-noxious stress (Rainero et al., 2001), etc. Some studies have shown different changes in low-frequency power (e.g., delta and theta frequency bands) (Bjork et al., 2009a, 2011b; Ojha and Panda, 2022) ADDIN. The relationship between various frequency bands and clinical characteristics of migraine is also explored in the studies (Bjork et al., 2009a,b, 2011b). The differences in specific frequency bands in migraineurs may depend on where on the scalp the abnormalities appear, in which phases of migraine the EEG signals were recorded and whether the responses were recorded at rest or were stimulus-evoked (Chamanzar et al., 2021). Although with heterogeneous results, most found differences are related to lower-frequency responses. Therefore, we believe that activities with lower frequencies (delta/theta/alpha) may be more suitable to be the characteristic frequency band for migraine recognition. Visual evoked potentials from 20 healthy controls and 70 migraine patients were analyzed in the frequency domain, and the author found that compared to healthy controls, interictal migraine patients had increased visually induced low frequency activity (Lisicki et al., 2020). The research of Gomez-Pilar et al. also confirmed our consideration and supports another fact that characteristic neural dynamics in migraine are linked to specific frequency bands but are not necessarily equal to conventional ones (Chamanzar et al., 2021).

Gomez-Pilar et al. also provided an additional band between 24.1 and 29.8 Hz to discriminate between migraine subgroups [chronic (CM) and episodic (EM)] (Chamanzar et al., 2021). A systematic review explored potential biomarkers to differentiate chronic and episodic migraine and proposed that future studies based on EEG should pay special attention to brain activity in medium and fast frequency bands, mainly the beta band (Gomez-Pilar et al., 2022). We find that the comparative study of migraine subtypes is more focused on migraine with aura and migraine without aura and more on the analysis of EEG signals under different stimulations. The different patterns of brain connectivity and networking observed in the two forms of migraine can be linked with the phenotypical differences in the perception of aura symptoms. Although no consensus regarding brain signatures for migraine and the subgroups has been reached, lack of consideration of migraine subgroups could also hide migraine general features due to the different behaviors of both subgroups in specific frequency bands (Gomez-Pilar et al., 2022). Based on the information above, we emphasize the importance of discriminating between migraine subgroups.

### 4.2. EEG features of the attack-initiating of migraine

By analyzing the EEG recorded in some phases or the whole cycle of migraine, the researcher found that neurophysiological changes seem to increase when a migraine attack approaches (Nyrke et al., 1990). Such changes in brain dynamics could have implications for understanding the complex neurophysiology of migraine before a headache attack. However, the results are diverse, and the findings of these studies include slow and asymmetric EEG activity before the attack, power decrease or increase in different frequency bands, frequency variability increased, connectivity reduced, and functional network separation, EEG phase coherence fluctuated across migraine phases (Cao et al., 2016), etc. A possible reason for the discordant results may be different timing of the recordings in relation to the migraine attacks. Some studies compared the pre-ictal and/or post-ictal interval with an interictal and/or ictal interval, and the studies used relatively different intervals (24–96 h) in the definition of the pre(post)-ictal phase. Another possible reason is the methodological difference. Some studies compared the pre-ictal recordings with the post-ictal ones, not with an interictal baseline interval as other studies did. To summarize, EEG-based brain state monitoring can identify physiological changes preceding a migraine attack, enabling valuable presymptom prediction and subsequent early intervention.

### 4.3. Migraine sensory processing

After reviewing the relevant EEG studies of migraine, we found that on the basis of existing EEG research, researchers used evoked potentials combined with QEEG to study migraine brain function changes under various stimuli, including visual stimulation, auditory stimulation, sound stimulation, olfaction stimulation, pain stimulation, etc. The number of studies on various EEG-derived evoked potentials is increasing, including visual evoked potentials (VEP), auditory evoked potentials (AEP), somatosensory evoked potentials (SEP), and event-related potentials (ERP) related to various tasks, for example, the “contingent negative variation” (CNV) (Meyer et al., 2016).

These studies are based on the characteristics of the lack of sensory habituation in migraine. However, the results of the studies have been discrepant. In a VEP-blinded study, the author believes that a lack of VEP habituation cannot be considered a reliable neurophysiological hallmark in migraine (Omland et al., 2016). Other study findings suggest that no evidence for a lack of habituation in any of the measures was seen between migraine patients and controls, and migraine patients process stimuli as more salient (Vila-Ballo et al., 2021). Ambrosini et al. (2017) found that VEP habituation was normal in healthy volunteers (HVs) and episodic migraine patients (EMs) during an attack but deficient in EMs interictally.

Between attacks, migraine patients are characterized by habituation of stimulation-evoked cortical responses. It is debated whether this is due to increased or decreased cortical excitability. Studies have concluded that deficient interictal pattern-reversal visual evoked potential (PR-VEP) habituation in migraine is due to a reduced, and not to an increased, pre-activation excitability level of the visual cortex (Bohotin et al., 2002). Additional findings suggest that the migraine brain displays abnormal visual evoked responses between migraine attacks. In migraine eyes, scotopic cone and rod responses increased. The results of this study support the hyperexcitability of the retina and cortex in patients with migraine (Mehnert et al., 2019). However, insufficient published data and substantial heterogeneity between studies were observed for all latency components of PR-VEPs, highlighting the need for further electrophysiological experimentation and more targeted temporal analysis of visual function in episodic migraineurs (Sezai et al., 2022). However, abnormal cortical responsivity and sensory processing may constitute the fingerprint of the migraine brain.

### 4.4. Research methods of migraine based on EEG

#### 4.4.1. Spectral power analysis

Concerning multichannel EEG, the frequency component is one of the most critical features. An EEG frequency spectrum was obtained with fast Fourier transformation (FFT) in many studies of migraine. Although FFT is commonly used in the data analysis process, and it works effectively for stationary signals, it has the problems of lower resolution and inherent “leakage” effects (Muthuswamy and Thakor, 1998). This shortcoming has motivated researchers to develop novel procedures and methods for spectral estimation, such as the Fourier decomposition method (Singh et al., 2017), variational mode decomposition (VMD) method (Liu et al., 2018), and Hilbert-Huang transform (HHT) method (Valderrama, 2021).

A small number of studies use the wavelet transform (WT). The wavelet has good time-frequency localized properties and multi-resolution analysis where the transient information of an EEG signal can be extracted efficiently (Daud and Sudirman, 2022). This method has good performance in the spectral analysis of irregular and non-stationary signals within different size windows (Al-Fahoum and Al-Fraihat, 2014). However, the WT suffers from Heisenberg uncertainty, which negatively affects its performance. Wavelet transforms offer certain advantages over fast Fourier transform techniques for the analysis of EEG (Akin, 2002).

#### 4.4.2. Functional connectivity and brain networks analysis

Functional connectivity is different from the traditional approach that analyses each brain area of EEG lead location. Functional connectivity allows the detection of common temporal features of two even distant neural populations due to weak reciprocal interactions or the shared influence of a third variable (Friston, 2011). The brain networking analysis, based on the connectivity models, may represent a way to explain the brain functions and neurological disorders. It can add knowledge about the complex mechanism of migraine, as the different brain interconnections may be an epiphenomenon of the altered neuronal excitability affecting the migraine brain (de Tommaso et al., 2014). Identifying connectome-based markers for migraine is necessary for developing new interventions or optimizing diagnosis and treatments for migraine headaches, which may benefit from targeting brain networks or systems rather than single structures (Maleki and Gollub, 2016).

Although many methods have been developed to investigate the functional interactions between brain areas, most functional connectivity analysis studies in migraine have been performed by resting-state functional magnetic resonance imaging (fMRI) (de Tommaso et al., 2021). Unlike functional magnetic resonance imaging, resting-state functional connectivity analysis of electrophysiological recordings can reveal intrinsic oscillatory and dynamic characteristics. EEG-based studies have observed aberrant functional connectivity in patients with migraine. However, the general impression emerging from the studies is that very different and incomparable recording and analysis methods are used. The selection of EEG reference value, artifact processing and filtering, period selection, frequency band selection, and other methodological choices can significantly affect the final results of functional connectivity or network research. Therefore, it is crucial to understand the characteristics of various measurement and analysis methods and make reasonable choices in research.

#### 4.4.3. Machine learning and deep learning

Machine learning approaches are promising techniques that allow the identification of possible biomarkers that could be used for early diagnosis, treatment planning, and monitoring of disease. It is largely used to develop automatic predictors in migraine classification.

Ferroni et al. explored an automated predictor to estimate medication overuse (MO) risk in migraine. The system combines support vector machines and random optimization (RO-MO). Receiver operating characteristic (ROC) analysis resulted in a c-statistic of 0.83 with a sensitivity and specificity of 0.69 and 0.87, respectively (Ferroni et al., 2020).

A study used machine-learning techniques to develop discriminative brain-connectivity biomarkers from resting-state functional magnetic resonance neuroimaging (rs-fMRI) data that distinguish between individual migraine patients and healthy controls. The author found that migraineurs with a longer disease burden were classified more accurately than migraineurs with a shorter disease burden (Chong et al., 2017). Another machine learning study based on magnetic resonance imaging (MRI) proposed a classification approach to examine whether the integration of multiple MRI features could improve the classification performance between migraine patients without aura (MWoA) and healthy controls. The final classification accuracy obtained was 83.67% (with a sensitivity of 92.86% and a specificity of 71.43%). It shows a promising classification capability for migraine by integrating information from multiple MRI features (Zhang et al., 2016).

To identify and validate the neural signatures of resting-state oscillatory connectivity for chronic migraine (CM), a classification model that employed a support vector machine was developed using magnetoencephalographic data to assess the reliability and generalizability of CM identification. The classification model exhibited excellent performance in distinguishing patients with CM from HCs (accuracy ≥ 86.8%, area under the curve (AUC) ≥ 0.9) and from those with episodic migraine (EM) (accuracy: 94.5%, AUC: 0.96). The model also achieved high performance (accuracy: 89.1%, AUC: 0.91) in classifying CM from other pain disorders (Hsiao et al., 2022).

A voxel-based machine learning analysis used fMRI to identify biomarkers to discriminate migraineurs as well as select patients suitable for transcutaneous vagus nerve stimulation (tVNS) treatment. By machine learning, two potential biomarkers were identified with an accuracy of 79.3%, sensitivity of 78.6%, and specificity of 80.0% (Fu et al., 2022).

These results indicate that electrophysiological and neuroimaging recordings in combination with machine learning can aid in individualized migraine diagnosis and prognosis. It greatly supports the diagnosis of clinical experts. However, we cannot exclude the possibility that the use of different data acquisition methods and acquisition protocols to study patients with different clinical conditions might have influenced the performance of the classification model. New classification studies with a larger sample size and multiple approaches should be conducted to identify the main differences in patients. On the other hand, the data consumed massive amount on storage media. Future research is still needed to train and test of this momentous volume of data, robustly evaluate algorithms and improve interpretability, generalizability and transparency. If such challenges can be overcome, machine learning has the potential to profoundly change the management of patients with migraine. With the rapid development of machine learning, various algorithms and classified distributions have emerged. In the future, we think higher accuracy rates might be obtained by combining electrophysiological and neuroimaging data and integrating multiple features.

## 5. Conclusion, challenges, and perspectives

Overall, existing EEG studies in migraine were heterogeneous and limited in terms of grouping, spatial undersampling (position and number of electrodes), data acquisition, and unstandardized analysis methods between studies. All of the above make it difficult to compare the results of different studies, and the findings are sometimes difficult to understand and rarely replicated. Nevertheless, these studies are instrumental in providing initial evidence of complex brain dysfunction in migraine. These electrophysiology changes provide hope to identify novel EEG biomarkers that can be targeted for migraine diagnosis and intervention and to understand the pathophysiology of migraine.

Future studies of migraine based on EEG should give specific attention to brain activity in lower-frequency bands. The migraine subtypes should be taken into account. Beyond demonstrating EEG changes in a frequency band, knowing in which cortical regions these changes are located is a major challenge in defining and understanding migraine biomarkers. In addition, we propose that future research should strictly focus on methodology and carry out repeated verification of multiple methods.

## Author contributions

YP and NZ made substantial contributions to conception and design. NZ wrote the manuscript. QZ, QC, YHu, NL, TS, and YL collected the materials. QY, SC, LH, YHo, and HL conducted the drawings. YP and NZ proposed key revisions to the manuscript. All authors contributed to this article and approved the submitted version.
